# Application of surgical gloves in cervical and scapular skin grafting surgery: A case report and literature review

**DOI:** 10.1097/MD.0000000000048258

**Published:** 2026-04-10

**Authors:** Huasheng Liu, Wenhui Xi, Huanli Wang, Tong Zhu

**Affiliations:** a Department of Burn Plastic and Cosmetic Surgery, Beijing Jishuitan Hospital Liaocheng Hospital, Liaocheng City, China; b Department of Burn Plastic and Cosmetic Surgery, Liaocheng People’s Hospital, Liaocheng City, China.

**Keywords:** case report, cervical region, scapular region, skin grafting, surgical gloves

## Abstract

**Rationale:**

Securing and protecting the transplanted skin grafts in the cervical and scapular regions posttransplantation is challenging.

**Patient concerns:**

A 42-year-old male was admitted for the treatment of burns in the neck and scapular regions. Due to the large size of the burn wounds in these areas, combined with the fact that a tracheostomy and intubation had already been performed, securing the skin grafts was challenging. During the first surgery, a tie-over dressing fixation method was used, but the survival rate of the transplanted skin was suboptimal.

**Diagnoses:**

The patient was diagnosed with third-degree burns in the neck and scapular regions, requiring surgical skin grafting for reconstruction.

**Interventions:**

Surgical gloves were used to secure and protect the skin grafts during cervical and scapular skin grafting. After debridement and skin grafting of the burn wounds in these areas, multiple layers of sterile gauze were applied over silver-containing dressings. Surgical gloves, cut and applied in a “shingle-like” fashion, were tensionally wrapped over the gauze. The edges of the gloves were sutured to the surrounding healthy skin to ensure proper fixation.

**Outcomes:**

When the dressing was removed 3 days later, the skin grafts were found to be well-fixed and viable.

**Lessons:**

Surgical gloves, being readily available, easily modifiable, and simple to apply, offer significant benefits for securing skin grafts in challenging areas such as the cervical and scapular regions.

## 1. Introduction

Skin grafting is a common technique for wound closure in burn surgery.^[1]^ Multiple factors influence the survival of transplanted skin grafts, with adequate fixation being paramount to ensure complete contact with the wound surface and prevent complications such as hematoma and serous fluid accumulation.^[2]^ Therefore, finding an ideal method for dressing fixation remains a critical area of research in burn surgery.^[3]^

After skin grafting in the cervical and scapular regions, protecting the surgical site is notably challenging. The high mobility of these areas often leads to the loosening of standard pressure dressings, which can result in displacement and detachment of the skin grafts.^[4]^ Furthermore, if the patient has undergone a tracheostomy, the presence of a tracheal tube secured with fixation ropes around the neck further complicates the stabilization of the cervical skin graft postoperatively.^[5]^

There are no clinical reports of their use in the cervical and scapular regions, to our knowledge. This case report details a patient with burns in these areas who, after unsatisfactory results with the traditional tie-over dressing, successfully used surgical gloves for fixation, resulting in effective survival of the skin grafts and healing of the burn wounds.

## 2. Case presentation

A 42-year-old male was admitted for the treatment of burns in the neck and scapular regions sustained. A prophylactic tracheostomy and intubation procedure were performed at another hospital due to significant swelling in the post-injury area of the neck. This factor further contributed to difficulties in securing the skin grafts in the neck and scapular regions after the transplantation.

The ethical approval is not applicable. The authors’ institution does not require ethical approval for reporting individual cases. The patient was informed that his medical record would be used for print and electronic case reports, and he agreed and provided written informed consent.

Upon admission to our hospital, the patient underwent debridement and dressing changes for his burn wounds, along with antibiotic treatment for infection. On the 9th day after the injury, the patient underwent his first debridement and skin grafting surgery. A tie-over dressing fixation method was initially used. During the procedure, large sheets of skin with open perforations were sutured together as a single unit and fixed to the neck and scapular wound areas. A silver-containing antimicrobial dressing was applied over the grafts. The tie-over dressing method was then used to secure the grafts in place, minimizing the risk of graft displacement. However, the survival rate of the transplanted skin was found to be suboptimal.

**Figure 1.**
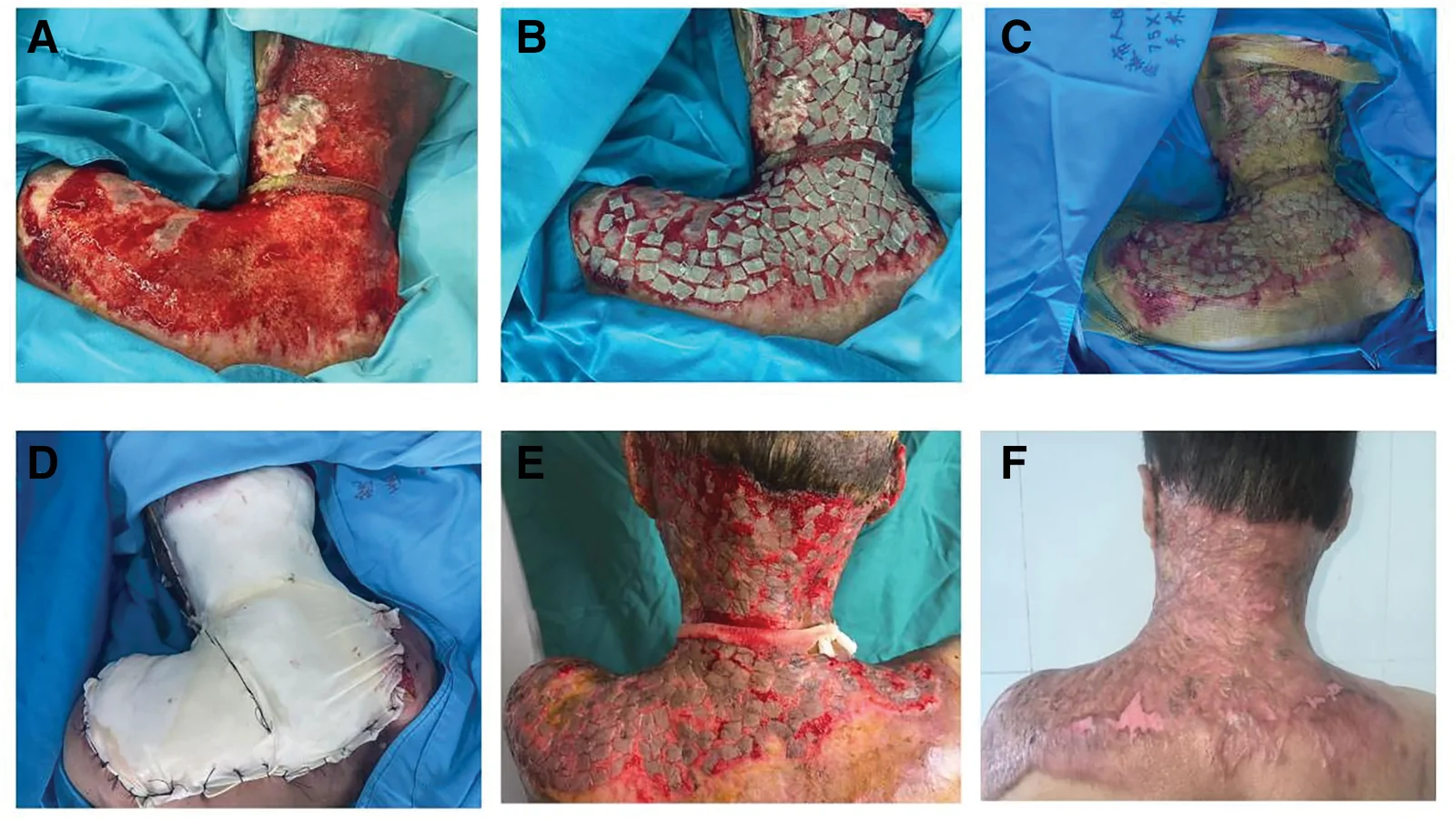
A 42-yr-old male patient admitted for flame burns on the neck. (A) Appearance of the neck and scapular wounds post-debridement; (B) posttransplantation status during surgery; (C) posttransplantation, skin grafts secured with a silver-antimicrobial dressing; (D) sterile gauzes placed over the silver-antimicrobial dressing, secured with a surgical glove; (E) on the third day postoperation, grafts on the neck and scapular wounds demonstrate good survival. (F) One month post-discharge, the patient returns for an outpatient visit, showing completely healed wounds.

**Figure 2.**
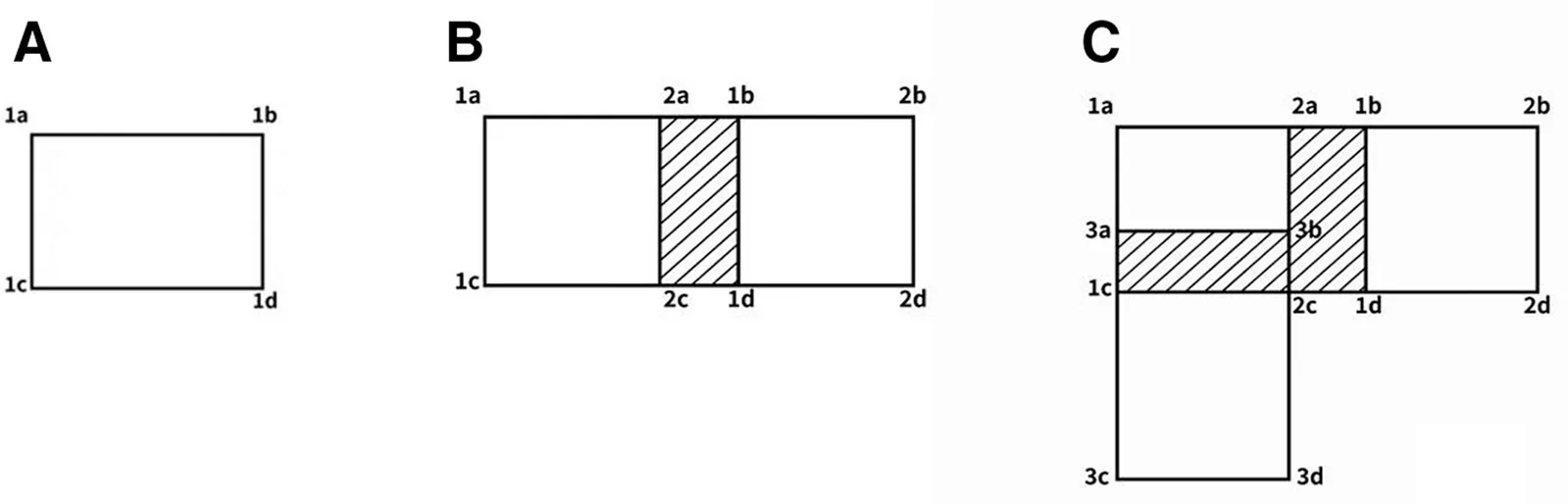
Schematic of the “shingle-like” coverage method using surgical gloves: 3 entire pieces of surgical glove materials are labeled 1, 2, and 3, with each corner marked by letters a, b, c, and d, such as 1a, 1b, 1c, 1d. The shaded areas between 2a2c and 1b1d represent the overlapping regions between the first and second pieces. (A) Surgical glove material labeled 1; (B) diagram showing overlap between glove materials 1 and 2; (C) diagram showing overlap between glove materials 1 and 3.

The patient’s wound bacterial culture revealed the presence of Enterobacter cloacae and Acinetobacter baumannii, sensitive to cefoperazone-sulbactam. Based on these results, cefoperazone-sulbactam was administered for anti-infection treatment. After over 2 weeks of wound dressing changes and topical application of mupirocin ointment, the wound exhibited no purulent discharge, fresh granulation tissue, and no bacterial growth. On the 27th day post-injury, a second debridement and skin grafting surgery were performed (Fig. 1A and B). The specific steps were as follows: (1) Split-thickness skin, taken from the head and measuring 1/100 inch thick, was cut into small skin grafts each approximately 0.5 cm × 0.5 cm in size. After debridement, hemostasis, and repeated washing of the wound with solutions including 0.9% saline, 3% hydrogen peroxide, iodophor with 0.5% effective iodine content, and again 0.9% saline, the small skin grafts were transplanted onto the wound. (2) A single layer of silver-containing dressing was placed over the transplanted skin grafts. Then, 3-0 sutures were used to perform interrupted stitches along the 4 edges of the silver-containing dressing to fix it to the surrounding normal skin, to prevent the skin grafts from shifting during subsequent operations (Fig. 1C). The silver-containing dressing selected for this case was produced by Shenzhen Yuanxing Pharmaceutical Company Limited, China. It is known as the Silver Sulfate Gauze Oil Dressing and consists of silver sulfate, medical-grade cotton fabric, and dimethylsilicone oil. The product is sterilized through irradiation and is intended for single use only. A silver-containing dressing with relatively large meshes was selected, which can not only resist infection but also facilitate the drainage of exudate. (3) Multiple layers of sterile gauze were stacked on the surface of the silver-containing dressing. A cut surgical glove was then placed over the gauze, with the rubber edge of the glove extending 1 cm beyond the edge of the post-graft wound (Fig. 1D). (4) The rubber edge of the glove’s “ab” side was fixed to the normal skin at the top of the wound edge with 3-0 sutures using interrupted stitches. (5) The “cd” side was pulled towards the bottom of the wound, and the “cd” side was sutured and fixed to the normal skin at the bottom of the wound edge. (6) The “ac” side was pulled towards the left of the wound, and the “ac” side was sutured and fixed to the normal skin at the leftmost edge of the wound. (7) The “bd” side was pulled towards the right of the wound, and the “bd” side was sutured and fixed to the normal skin at the rightmost edge of the wound. (8) If the wound exceeded the size of 1 glove, multiple gloves were used, shingled according to the size and shape of the wound. The overlapping part between the second glove and the first was generally about 2 cm, aimed at increasing the tension in the overlap to prevent the gloves from shifting, which would undermine the pressure on the sterile gauze below (Fig. 2A–C). After overlapping multiple gloves, the fixation procedure remained consistent with steps (4) to (7) Finally, in order to enhance the breathability of the surgical gloves, a sharp blade can be used to pierce numerous small holes on the surface of the surgical gloves.

During the surgical procedure, we closely monitored the patient’s heart rate, blood pressure, and oxygen saturation, and observed indicators such as skin color, capillary refill time, pulse strength, and any signs of swelling or discomfort in the neck area to promptly assess pressure-related risks. Postoperatively, we continued to monitor the patient’s heart rate, blood pressure, oxygen saturation, and pulse strength, while closely observing for any symptoms such as dizziness, vertigo, or tenderness in the carotid sinus area, and promptly assessing pressure-related risks.

**Figure 3.**
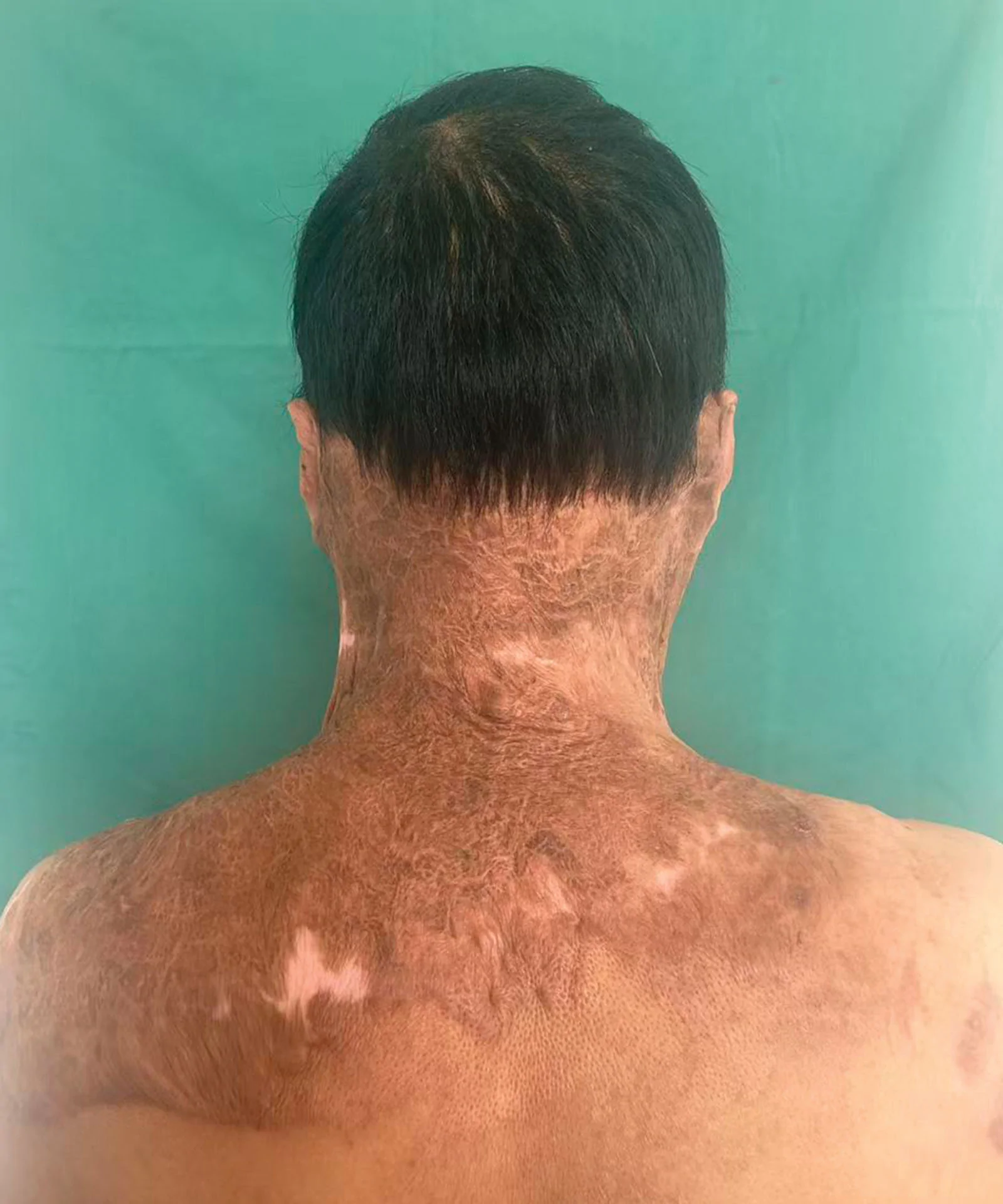
Photograph of 1-yr follow-up of the patient’s neck and scapular skin grafting.

Three days post-skin grafting surgery, the dressing was removed, revealing well-fixed, viable transplanted skin grafts (Fig. 1E). Eight days later, the wounds on the neck and scapular regions had generally healed, and the patient was discharged. One month post-discharge, the patient returned for a follow-up at the outpatient department, where complete healing of the wounds was confirmed (Fig. 1F). Regular telephone follow-ups were conducted within 1 year, and the patient reported being satisfied with their functional recovery, with no impact on daily life. At the 1-year mark, there was no significant scarring contracture in the patient’s neck (Fig. 3).

## 3. Discussion

After skin grafting, regions such as the neck, scapula, and buttocks pose challenges for fixation due to their extensive range of motion and frequent activity.^[6,7]^ Common fixation methods post-surgery include standard pressure dressings, tie-over dressing, and negative pressure wound therapy (NPWT) systems.^[8]^ Conventional pressure dressings, often used for limb grafts, are simple and cost-effective but may allow graft displacement during application.^[9]^ Tie-over dressing effectively secure the grafts, ensuring proper contact with the wound bed and preventing hematoma or seroma beneath the grafts, thereby promoting graft survival.^[10]^ However, these dressings have several drawbacks: they can cause inward rolling of the wound edges, leading to potential graft displacement at the margins^[11]^; they require cooperation from at least 2 people, involve lengthy procedures, and lead to suture wastage.^[12,13]^ For this patient, insufficient pressure may result in poor graft adherence, increasing the risk of hematoma or seroma, while excessive pressure can compress the trachea, impairing breathing, and potentially depress blood pressure by affecting the carotid sinus.^[14,15]^ Furthermore, sutures lack elasticity; after dressing, they become rigid. Any prolonged pressure or patient movement may stretch the sutures, causing them to cut into the skin and soft tissues, thereby loosening the anchoring end of the suture and compromising the fixation effectiveness.^[11]^ NPWT systems enhance graft survival, particularly in joints and over the scapula where conventional dressings fail to secure the grafts adequately. They create a stable negative pressure environment conducive to graft adherence.^[16]^ However, in the neck region, controlling pressure with NPWT techniques also presents challenges, similar to those encountered with tie-over dressing.^[17]^ Additionally, for primary healthcare facilities in low- and middle-income countries, NPWT systems are prohibitively expensive.^[16]^

The application of surgical gloves for securing skin grafts post-grafting has been reported in the literature. Mashiko et al demonstrated successful postoperative survival of skin grafts on the fingers using surgical gloves for fixation.^[18]^ Their technique ensured uniform pressure across the graft area, preventing hematoma or seroma formation, and maintaining graft adherence, especially in small, mobile areas like the fingers. Their study primarily focused on hand and finger burn patients. Similarly, Sahin et al described using surgical gloves for fixation after split-thickness skin grafting on the dorsal foot.^[19]^ They highlighted the ease of applying pressure with surgical gloves, which provides adequate fixation and helps prevent complications such as infection or graft displacement, particularly in small graft areas. In contrast, our approach, inspired by the overlay pattern of Chinese roof tiles, used a similar “shingle-like” method for securing skin grafts in the neck and scapular regions, which present greater anatomical challenges and a larger range of motion. Unlike previous studies focusing on smaller, immobilized areas, our method demonstrated the feasibility of using surgical gloves for larger, more complex regions. We found that the method provided sufficient pressure to secure grafts without causing hematoma, seroma, or graft displacement. It also avoided complications related to tracheal and carotid sinus compression, particularly in the neck, where excessive pressure can affect vital structures. The flexibility of the “shingle-like” coverage allowed for the use of multiple surgical gloves, accommodating larger wounds. This technique could also be applied to graft fixation in other trunk areas, including the anterior chest, lateral chest, abdomen, and scapular regions.

Surgical gloves are readily available and economical.^[20,21]^ Compared to NPWT systems, they offer a cost-effective alternative. The postoperative graft survival observed suggests the simplicity, reliability, and effectiveness of this method, making it a potentially valuable option for burn skin grafting in resource-limited settings. However, this study reports only 1 case, and conclusions drawn from a single case may not be widely applicable. Therefore, the aim of this study is to present a novel and practical technique for clinical reference. In the future, we plan to accumulate more cases and possibly conduct case-control studies to further investigate the reliability and evidence supporting this method.

## 4. Limits

The fixation method using surgical gloves has inherent disadvantages. As nonspecialized materials, they may not match the breathability and biocompatibility of dedicated medical fixation devices.^[7]^ Prolonged use could potentially alter the local skin microenvironment, increasing the risk of infection or allergic reactions.^[22]^ While perforating the gloves enhances breathability, it may compromise their effectiveness, especially during vigorous or frequent movements, potentially leading to loosening. Therefore, this method necessitates vigilant monitoring and timely adjustments by healthcare providers.^[18]^ One additional limitation should be addressed: we applied the graft as multiple units, rather than as a single-unit graft application. Single-unit graft applications are more prone to graft survival issues due to incomplete wound bed contact or dehiscence. In contrast, applying the graft as multiple units may provide better contact with the wound bed, reducing these issues. Furthermore, the generally convex surface of the graft may have positively contributed to graft survival, indicating that using multiple unit grafts, in some cases, provides better survival conditions, rather than solely the application of the surgical glove fixation method.

## 5. Conclusion

In summary, employing surgical gloves for skin graft fixation on the posterior neck and scapular areas is both innovative and practical. It addresses the limitations of conventional fixation methods and provides an effective alternative for these challenging regions. Nonetheless, it is important to acknowledge its limitations. Future research and clinical applications should focus on enhancing the material properties of surgical gloves to improve their breathability and biocompatibility, or integrating them with other fixation techniques to augment stability.

## Acknowledgments

The authors thank Dr Xiaohua Hu from the Department of Burn Plastic Surgery, Beijing Jishuitan Hospital, for his guidance in the patient’s treatment process and the editing of the first draft of this article.

## Author contributions

**Conceptualization:** Huasheng Liu, Tong Zhu.

**Data curation:** Huasheng Liu, Wenhui Xi, Huanli Wang, Tong Zhu.

**Formal analysis:** Wenhui Xi, Huanli Wang, Tong Zhu.

**Methodology:** Huasheng Liu, Wenhui Xi, Huanli Wang, Tong Zhu.

**Supervision:** Tong Zhu.

**Writing – original draft:** Huasheng Liu.

**Writing – review & editing:** Huasheng Liu, Tong Zhu.
